# International Medullary Thyroid Carcinoma Grading System: A Validated Grading System for Medullary Thyroid Carcinoma

**DOI:** 10.1200/JCO.21.01329

**Published:** 2021-11-03

**Authors:** Bin Xu, Talia L. Fuchs, Sara Ahmadi, Mohammed Alghamdi, Bayan Alzumaili, Mohamed-Amine Bani, Eric Baudin, Angela Chou, Antonio De Leo, James A. Fagin, Ian Ganly, Anthony Glover, Dana Hartl, Christina Kanaan, Pierre Khneisser, Fedaa Najdawi, Aradhya Nigam, Alex Papachristos, Andrea Repaci, Philip M. Spanheimer, Erica Solaroli, Brian R. Untch, Justine A. Barletta, Giovanni Tallini, Abir Al Ghuzlan, Anthony J. Gill, Ronald A. Ghossein

**Affiliations:** ^1^ Department of Pathology, Memorial Sloan Kettering Cancer Center, New York, NY; ^2^ NSW Health Pathology, Department of Anatomical Pathology, Royal North Shore Hospital, St Leonards, NSW, Australia; ^3^ University of Sydney, Sydney, NSW, Australia; ^4^ Department of Medicine, Division of Endocrinology and Metabolism, Brigham and Women's Hospital, Boston, Harvard Medical School, MA; ^5^ Medical Pathology and Biology Department, Gustave Roussy Campus Cancer, Villejuif, France; ^6^ Department of Endocrine Oncology and Nuclear Medicine, Gustave Roussy Cancer Campus Grand Paris, Villejuif, France; ^7^ Pathology Unit-Azienda USL di Bologna, Department of Experimental, Diagnostic and Specialty Medicine, University of Bologna, Bologna, Italy; ^8^ Division of Subspecialty Medicine, Memorial Sloan Kettering Cancer Center, New York, NY; ^9^ Department of Surgery, Memorial Sloan Kettering Cancer Center, New York, NY; ^10^ Endocrine Surgical Unit, Royal North Shore Hospital, St Leonards, NSW, Australia; ^11^ Department of Surgery, Gustave Roussy Cancer Campus Grand Paris, Villejuif, France; ^12^ Department of Pathology, Brigham and Women's Hospital, Boston, MA; ^13^ Endocrinology Unit, IRCCS Azienda Ospedaliero—Universitaria di Bologna, Bologna, Italy; ^14^ Endocrinology Unit-Azienda USL di Bologna, Bologna, Italy

## Abstract

**PURPOSE:**

Medullary thyroid carcinoma (MTC) is an aggressive neuroendocrine tumor (NET) arising from the calcitonin-producing C cells. Unlike other NETs, there is no widely accepted pathologic grading scheme. In 2020, two groups separately developed slightly different schemes (the Memorial Sloan Kettering Cancer Center and Sydney grade) on the basis of proliferative activity (mitotic index and/or Ki67 proliferative index) and tumor necrosis. Building on this work, we sought to unify and validate an internationally accepted grading scheme for MTC.

**PATIENTS AND METHODS:**

Tumor tissue from 327 patients with MTC from five centers across the United States, Europe, and Australia were reviewed for mitotic activity, Ki67 proliferative index, and necrosis using uniform criteria and blinded to other clinicopathologic features. After reviewing different cutoffs, a two-tiered consensus grading system was developed. High-grade MTCs were defined as tumors with at least one of the following features: mitotic index ≥ 5 per 2 mm^2^, Ki67 proliferative index ≥ 5%, or tumor necrosis.

**RESULTS:**

Eighty-one (24.8%) MTCs were high-grade using this scheme. In multivariate analysis, these patients demonstrated decreased overall (hazard ratio [HR] = 11.490; 95% CI, 3.118 to 32.333; *P* < .001), disease-specific (HR = 8.491; 95% CI, 1.461 to 49.327; *P* = .017), distant metastasis-free (HR = 2.489; 95% CI, 1.178 to 5.261; *P* = .017), and locoregional recurrence-free (HR = 2.114; 95% CI, 1.065 to 4.193; *P* = .032) survivals. This prognostic power was maintained in subgroup analyses of cohorts from each of the five centers.

**CONCLUSION:**

This simple two-tiered international grading system is a powerful predictor of adverse outcomes in MTC. As it is based solely on morphologic assessment in conjunction with Ki67 immunohistochemistry, it brings the grading of MTCs in line with other NETs and can be readily applied in routine practice. We therefore recommend grading of MTCs on the basis of mitotic count, Ki67 proliferative index, and tumor necrosis.

## INTRODUCTION

Medullary thyroid carcinoma (MTC) is a primary neuroendocrine carcinoma of the thyroid gland that arises from the calcitonin-secreting parafollicular C cells.^1,2^ Established prognostic factors conferring worse outcome include clinical and molecular characteristics, such as age, sex, TNM stage, sporadic versus hereditary disease, distant metastasis, nodal metastatic burden, serum calcitonin, serum carcinoembryonic antigen (CEA), type of somatic *RET* mutation, response to initial therapy, and extent of thyroidectomy.^3-7^ Unlike pulmonary and gastrointestinal neuroendocrine neoplasms, which have well accepted and validated histologic grading systems on the basis of mitotic count, Ki67 proliferative index, and assessment of tumor necrosis,^8,9^ there is currently no established grading scheme for MTC.

**CONTEXT**

**Key Objective**
Currently, there is no widely accepted prognostically relevant pathologic grading system for medullary thyroid carcinoma (MTC). In this study, we proposed and validated an international medullary thyroid carcinoma grading system (IMTCGS) using a retrospective cohort of 327 patients with MTC from five centers across the United States, Europe, and Australia.
**Knowledge Generated**
The IMTCGS, a two-tiered grading system that defines high-grade MTC as tumors with at least one of the following three features: mitotic index≥ 5 per 2 mm^2^, Ki67 proliferative index ≥ 5%, and/or tumor necrosis, is a robust independent prognostic tool for patients with MTC.
**Relevance**
The IMTCGS can identify MTC with a profoundly greater risk of progressive and fatal disease and can be included in risk stratification and clinical decisions for MTC.

Recently, two independent studies, one from Memorial Sloan Kettering Cancer Center (MSKCC, New York, NY) by Alzumaili et al^10^ and the other from Royal North Shore Hospital, Sydney, Australia, by Fuchs et al,^11^ independently identified mitotic index (MI), tumor necrosis, and/or Ki67 proliferative index as prognostic histologic features in MTC, and subsequently proposed two different but broadly similar histologic grading schemes for MTC. These schemes were both based on proliferative activity (MI and/or Ki67 proliferative index) and tumor necrosis, but used different cutoffs for prognostic categories. The MSKCC system is two-tiered and defined high-grade tumors as those having MI ≥ 5 per 2 mm^2^ and/or tumor necrosis. The Sydney grading scheme is three-tiered and defined low-grade tumors as those having MI < 3 mitoses per 2 mm^2^, a Ki67 proliferative index of < 3%, and no tumor necrosis; intermediate grade as an MI of 3-20 mitoses per 2 mm^2^ or a Ki67 proliferative index of 3%-20% without necrosis, or a low proliferative index with necrosis; and high grade as intermediate proliferative activity with necrosis, or high proliferative activity with or without necrosis.

Both schemes have been shown to have merit in their own right^10,11^ and were validated in subsequent independent cohorts.^12^ However, there would be a clear advantage in developing a universal grading scheme with consensus cutoffs for all indices that has been validated in multiple international centers. We therefore created an international MTC working group from five major centers with the goal of developing and validating an internationally accepted grading scheme for MTC.

## MATERIAL AND METHODS

### Study Cohort

The study was approved by the institutional review board of each participating site. This retrospective study included a total of 327 patients with resected MTC gathered from five academic centers (Royal North Shore Hospital, Sydney, Australia: n = 79; Institut Gustave Roussy, Villejuif, France: n = 70; MSKCC, New York, NY: n = 69; University of Bologna Medical Center, Bologna, Italy: n = 65; and Brigham and Women's Hospital, Boston, MA: n = 44). A proportion (n = 192) of the study cohort were included in prior studies, although all prognostic factors were independently assessed and survival data updated for this study.^10-12^ All slides were reviewed at individual participating sites by at least one specialist endocrine pathologist blinded to the patients' outcome.

### Clinicopathologic Review and Grading

The initial thyroidectomy specimen was examined microscopically (thyroid and accompanying lymph nodes) and grading was on the basis of the primary tumor. The MI and Ki67 proliferative index were evaluated using the same methods proposed for gastrointestinal neuroendocrine tumors (GINET)^9^ (Table 1). Briefly, both measurements were obtained in the area showing the highest proliferative activity (so called hotspots). MI was assessed per 2 mm^2^, equivalent to 10 high-powered fields in many microscopes in widespread use. For the Ki67 proliferative index, 500-2,000 tumor cells were counted per tumor.

**TABLE 1.**
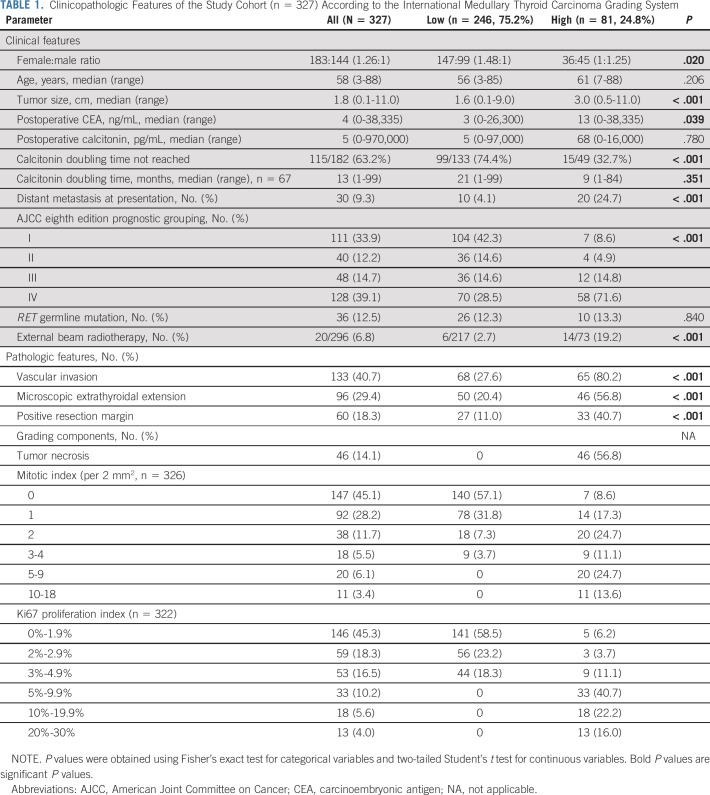
Clinicopathologic Features of the Study Cohort (n = 327) According to the International Medullary Thyroid Carcinoma Grading System

**FIG 1.**
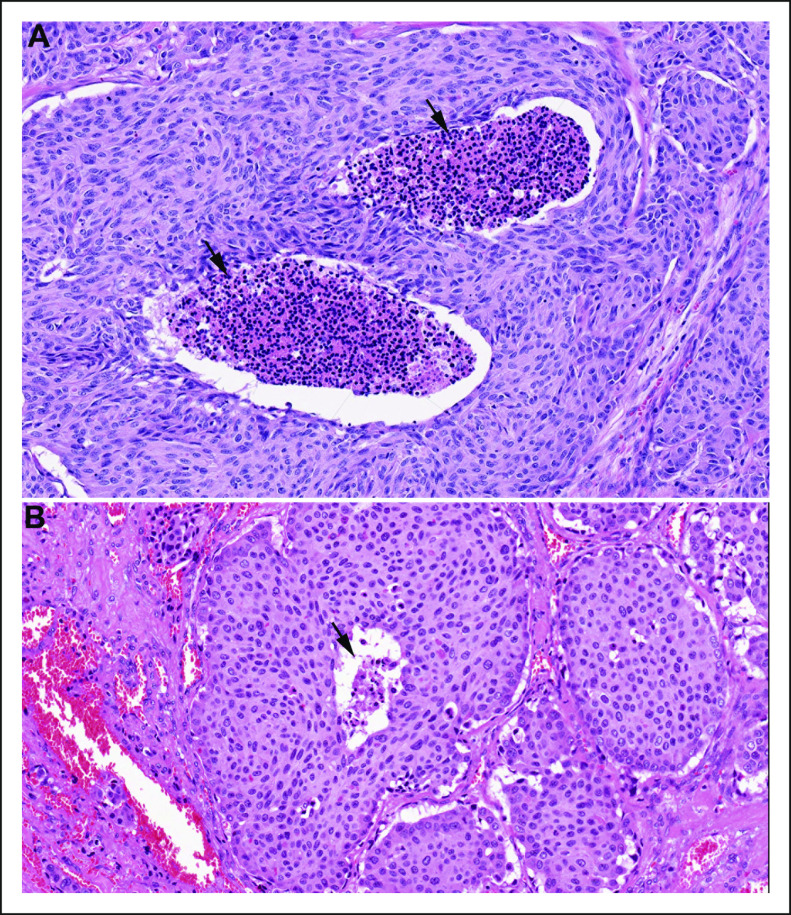
Tumor necrosis (arrows) in medullary thyroid carcinoma. Tumor necrosis can be prominent and (A) easily detected or (B) focal.

Necrosis was defined as tumor necrosis associated with degenerating cytoplasm and punctate karyorrhectic nuclear debris regardless of its extent (Fig 1). Infarct-like necrosis, which is usually associated with a fibroblastic stromal reaction, hemorrhage, or identifiable fine-needle aspiration tract, was not considered necrosis for the purposes of this study.^13^

Other established prognostic clinical and pathologic parameters that were collected included sex, age, tumor size, postoperative serum CEA, postoperative serum calcitonin, American Joint Committee on Cancer (AJCC) eighth edition prognostic stage groups, status of *RET* germline mutation, vascular invasion, microscopic extrathyroidal extension, and resection margin status.

### Outcome and Statistical Analysis

Three hundred and twenty-two patients had follow-up data available. The outcomes collected included overall survival (OS), disease-specific survival (DSS), locoregional recurrence-free survival (LRRFS), and distant metastasis-free survival (DMFS). All statistical analyses were performed using the SPSS software 24.0 (IBM Corporation, Armonk, NY). The correlation between histologic grade and various clinicopathologic parameters was calculated using Fisher's exact test for categorical variables and two-tailed Student's *t* test for continuous variables. The prognostic impact of each parameter on OS, DSS, DMFS, and LRRFS was calculated using univariate Cox proportional hazard regression models for continuous variables and log-rank tests for categorical variables. Factors significant on univariate analysis were subsequently subjected to multivariate analysis using Cox proportional hazards model. *P* values < .05 were considered to be statistically significant.

### Determination of a Consensus Grading Scheme

In addition to the Sydney and MSKCC grading schemes, eight other potential grading schemes (four two-tiered and four three-tiered) were investigated using various cutoffs and combinations of the key pathologic prognostic factors of MI, Ki67, and tumor necrosis (Data Supplement, online only). A consensus conference was subsequently held, and a single system, hereby termed the International Medullary Thyroid Carcinoma Grading System (IMTCGS), was agreed upon and investigated in detail. This scheme is presented in the Data Supplement. In brief, an MTC was considered as high grade when it had at least one of the following three features: MI ≥ 5 per 2 mm^2^, Ki67 proliferative index ≥ 5%, and/or tumor necrosis.

## RESULTS

### Clinicopathologic Features of the Study Cohort

The clinicopathologic characteristics of the study cohort are shown in Table 1. The median age was 58 years (range, 3-88 years). There was a female predominance with a female:male ratio of 1.26:1. The AJCC eighth edition prognostic stage groups were distributed as follows: stage I 33.9%, stage II 12.2%, stage III 14.7%, and stage IV 39.1%. Thirty-six patients (36 of 287, 12.5%) were known to have germline *RET* mutations and therefore had multiple endocrine neoplasia type 2 syndrome. Thirty patients (9.3%) had distant metastasis at presentation. Postoperative serum CEA and calcitonin level were available in 194 and 281 patients, respectively, with a median of 4 ng/mL (range, 0-38,335 ng/mL) and 5 pg/mL (range, 0-970,000 pg/mL), respectively. Calcitonin doubling time was available in 182 patients. Among them, calcitonin levels did not double during follow-up in 115 (63.2%) patients. Histologic evidence of vascular invasion, microscopic extrathyroidal extension, and positive resection margins were identified in 113 (40.8%), 96 (29.5%), and 59 (19.1%) patients, respectively.

### Histologic Features Used in the Grading Schemes

The number and percentage of MTC according to the histologic features included in the grading scheme (namely MI, Ki67 proliferative index, and tumor necrosis) are presented in Table 1. In the entire study cohort, 277 (85.0%), 295 (90.5%), and 315 (96.6%) cases had an MI of < 3, < 5, and < 10 per 2 mm^2^, respectively. There were no cases with an MI > 20 per 2 mm^2^. The number of tumors with a Ki67 proliferative index of < 3%, 3%-20%, and > 20% was 205 (63.7%), 106 (32.9%), and 11 (3.4%), respectively. Tumor necrosis was identified in 46 MTCs (14.1%).

All of the individual histologic features used for the grading scheme, including MI per 2 mm^2^ (< 5 *v* ≥ 5, or < 3 *v* 3-20), Ki67 proliferative index (< 3%, 3%-20%, and > 20%), and tumor necrosis, were significant predictors of OS, DSS, LRRFS, and DMFS, with the exception of Ki67 proliferative index < 3% versus > 20% for DSS (*P* = .298, Table 2). The Kaplan-Meier survival curves for DSS stratified by necrosis, MI, and Ki67 proliferative index are provided in the Data Supplement.

**TABLE 2.**
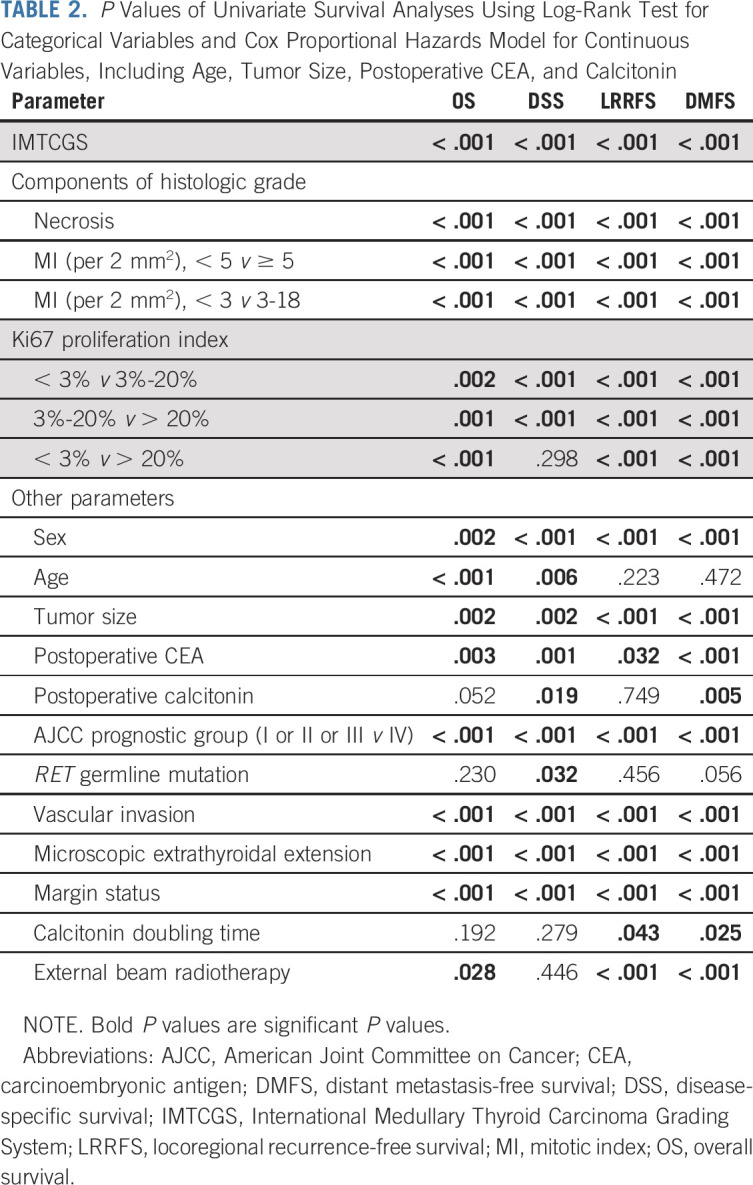
*P* Values of Univariate Survival Analyses Using Log-Rank Test for Categorical Variables and Cox Proportional Hazards Model for Continuous Variables, Including Age, Tumor Size, Postoperative CEA, and Calcitonin

### Determination of an IMTCGS

The Kaplan-Meier survival curves and the results of multivariate survival analyses for the MSKCC, Sydney, and eight other grading schemes that were trialed are provided in the Data Supplement.

After reviewing all relevant results and evaluating the prognostic significance of each individual grading system, a single grading scheme, the IMTCGS, was endorsed at a consensus conference attended by pathologists from all sites (B.X., T.L.F., A.A., J.A.B., G.T., A.J.G., and R.A.G.). The IMTCGS is a two-tiered system in which low-grade MTCs are defined as tumors with an MI of < 5 mitoses/2 mm^2^, a Ki67 proliferative index of < 5%, and absence of tumor necrosis, whereas high-grade MTCs are those with at least one of the following three features: MI ≥ 5 mitoses per 2 mm^2^, Ki67 proliferative index ≥ 5%, or tumor necrosis (Data Supplement). Using this consensus grading scheme, 246 MTCs (75.2%) were classified as low grade and 81 (24.8%) were classified as high grade.

### Clinicopathologic Characteristics According to Histologic Grade

Correlations between the IMTCGS grade and various clinicopathologic features are illustrated in Table 1. Compared to those with low-grade MTCs, patients with high-grade tumors were more commonly male; had larger tumors; had higher postoperative CEA; were more likely to have distant metastasis at presentation; were more frequently AJCC eighth edition prognostic stage group IV; were more likely to have vascular space invasion, microscopic extrathyroidal extension, and positive resection margins; were more likely to be offered external beam radiotherapy; and had a lower serum calcitonin doubling time (*P* < .05). Other features did not differ significantly among MTCs of different histologic grades.

### Validation of the Prognostic Value of the IMTCGS

**FIG 2.**
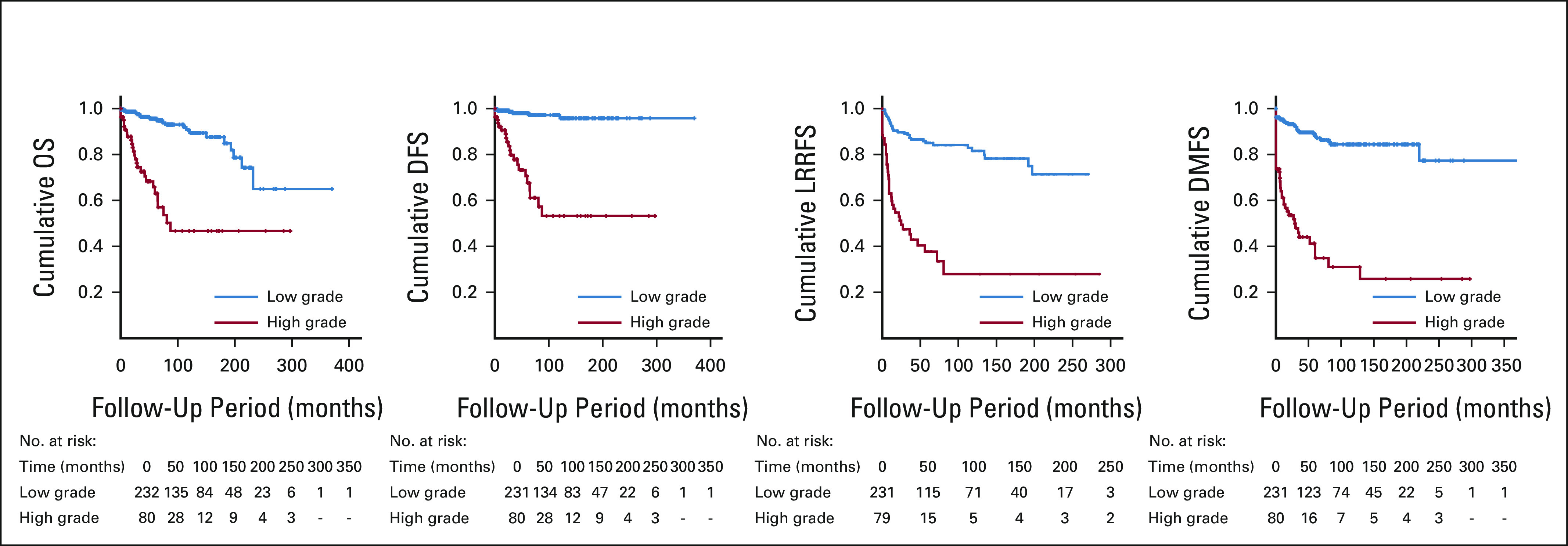
Kaplan-Meier curves for survival according to the international medullary thyroid carcinoma grading system. DMFS, distant metastasis-free survival; DSS, disease-specific survival; LRRFS, locoregional recurrence-free survival; OS, overall survival.

Among the study cohort, 322 patients had follow-up data available, with a median follow-up period of 55 months (range, 0-370 months). The 3-, 5-, and 10-year OS, DSS, LRRFS, and DMFS for the entire cohort stratified according to histologic grade are presented in Table 3. On univariate analyses, the IMTCGS was a significant prognostic indicator for OS, DSS, DMFS, and LRRFS (*P* < .001, Table 3). For IMTCGS low-grade versus high-grade, OS (95% CI) was 96% (94 to 99%), 96% (93 to 99), and 91% (85 to 96) versus 73% (62 to 84), 66% (54 to 78), and 47% (31 to 63) at 3 years, 5 years, and 10 years, respectively. DSS was 98% (96 to 100), 98% (96 to 100), and 97% (94 to 100) versus 78% (67 to 88), 71% (58 to 83), and 53% (37 to 70); DMFS was 90% (86 to 94), 88% (84 to 93), and 84% (78 to 90) versus 44% (32 to 56), 41% (28 to 53), and 31% (17 to 44); and LRRFS was 89% (84 to 93), 85% (80 to 90), and 82% (75 to 88) versus 47% (35 to 60), 37% (24 to 50), and 28% (13 to 43), respectively. The Kaplan-Meier survival curves are shown in Figure 2.

**TABLE 3.**
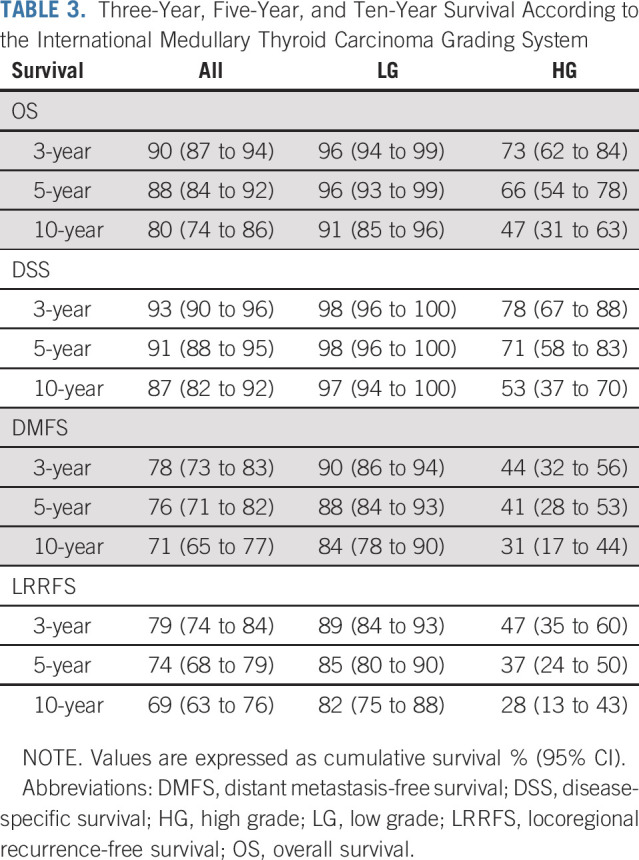
Three-Year, Five-Year, and Ten-Year Survival According to the International Medullary Thyroid Carcinoma Grading System

Other clinicopathologic characteristics that were prognostically significant for OS, DSS, DMFS, and LRRFS included age, sex, tumor size, postoperative CEA, AJCC eighth edition prognostic stage group, vascular invasion, microscopic extrathyroidal extension, and margin status. Greater age was associated with worse OS and DSS. The provision of external beam radiotherapy was associated with a worse outcome for OS, DMFS, and LRRFS. A shorter calcitonin doubling time was associated with worse for DMFS and LRRFS. Higher postoperative serum calcitonin and the presence of *RET* germline mutation were associated with shorter DSS (Table 2).

Multivariate survival analyses using Cox proportional hazard regression models demonstrated that high-grade MTC defined using the IMTCGS was an independent predictor of decreased OS (hazard ratio [HR] = 10.847; 95% CI, 2.903 to 40.531; *P* < .001), DSS (HR = 8.491; 95% CI, 1.461 to 49.327; *P* = .017), DMFS (HR = 2.267; 95% CI, 1.018 to 5.049; *P* = .045), and LRRFS (HR = 1.938; 95% CI, 1.044 to 3.876; *P* = .042; Table 4). The prognostic significance of the IMTCGS was maintained in subgroup analyses of the separate cohorts from each of the five centers (Data Supplement).

**TABLE 4.**
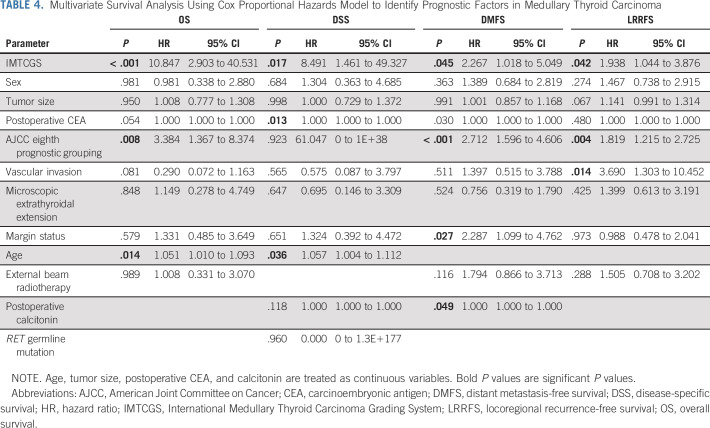
Multivariate Survival Analysis Using Cox Proportional Hazards Model to Identify Prognostic Factors in Medullary Thyroid Carcinoma

Other independent prognostic factors identified on multivariate survival analyses included age for OS and DSS; AJCC eighth edition prognostic stage group for OS, LRRFS, and DMFS; postoperative serum CEA for DSS; postoperative serum calcitonin and margin status for DMFS; and vascular invasion for LRRFS. The HR, 95% CI, and *P* values for all parameters analyzed in multivariate survival analyses are presented in Table 4.

## DISCUSSION

There is now broad agreement that neuroendocrine tumors (NETs) of many sites can be graded on the basis of the simple factors of mitotic count and Ki67 proliferative index, with the addition of tumor necrosis for NETs arising in certain sites such as pulmonary carcinoids.^8,9^ The IMTCGS therefore brings the grading of MTC in line with the grading of other NETs. In this study, we provide firm evidence that the simple two-tiered IMTCGS system, on the basis of MI, Ki67 proliferative index ≥ 5%, and tumor necrosis, is highly predictive of overall, disease-specific, distant metastasis-free, and locoregional recurrence-free survival in MTC.

Necrosis is a binary variable. That is, tumors either have necrosis or do not, and the presence of any true tumor necrosis is sufficient for classification of an MTC as high grade. In fact, in this cohort, the 3-year, 5-year, and 10-year DSS for MTCs without tumor necrosis was 97%, 97%, and 95%, respectively, and for MTCs with tumor necrosis was 65%, 56%, and 34%, respectively. Furthermore, as shown in the Data Supplement, MTCs with tumor necrosis, regardless of MI and Ki67 proliferative index, were associated with decreased DSS.

However, we emphasize that both MI and Ki67 proliferative index are continuous variables, and as each variable increases, prognosis worsens (Data Supplement). We therefore strongly recommend documentation of the precise mitotic count and Ki67 index in addition to the overall histologic grade in the pathology reports of MTC, in a manner analogous to NETs of other organs. Although accepting that MI and Ki67 are continuous variables, to stratify large populations into distinct risk groups, there is a need for specific cutoffs. The WHO classification (fifth edition, 2020) divides well-differentiated GINET into three grades based solely on MI and Ki67 proliferative index, using cutoffs of < 2, 2-20, and > 20 per 2 mm^2^ for MI, and < 3%, 3%-20%, and > 20% for Ki67 index.^9^ The WHO classification (fifth edition, 2021)^14^ subdivides low-grade pulmonary neuroendocrine neoplasms (that is pulmonary carcinoids) into typical carcinoid with MI < 2 per 2 mm^2^ and lacking necrosis, atypical carcinoid with 2-10 mitoses per 2 mm^2^ and/or necrosis, and neuroendocrine carcinomas comprising small cell carcinoma and large cell neuroendocrine carcinoma with an MI > 10 per 2 mm^2^. Although it would be ideal to unify grading systems across all NETs of all body sites with identical cutoffs,^15^ our data indicate that subdividing MTC into prognostically significant risk groups requires different cutoffs. For example, the cutoffs used for grade 3 GINET (ie, MI > 20 per 2 mm^2^ or Ki67 proliferation index > 20%) would be impractical as very few MTCs fall into this category. In fact, no tumor in the entire cohort had an MI of > 20 per 2 mm^2^ and only 11 MTCs (3.4%) had a proliferation index of > 20%. Similarly, the overwhelming majority (319 of 326, 97.9%) of MTCs in our cohort showed a mitotic count of ≤ 10 per 2 mm^2^, the MI cutoff used to define neuroendocrine carcinoma in the lung. We trialed several different systems with different cutoffs, presented in the Data Supplement, and concluded that the IMTCGS two-tiered system that defined high-grade MTCs as tumors with at least one of the following features: MI ≥ 5 per 2 mm^2^, Ki67 proliferative index ≥ 5%, or tumor necrosis, was optimal. Although each of the other potential schemes were prognostic to some degree, illustrating the continuous nature of MI and Ki67 index, in the three-tiered systems, the intermediate-grade MTCs generally lacked prognostic significance in multivariate survival analyses when compared with low-grade MTCs, but maintained significance when compared with high-grade tumors. Therefore, a two-tiered grading scheme was preferred. Using our preferred cutoffs, only 81 (24.8%) MTCs were considered high grade, but these tumors had significantly shorter OS of only 47% at 10 years versus 91% for their low-grade counterparts.

Histologic grading is only one component of patient risk assessment and, similar to previous studies,^3,6,16-20^ we demonstrate that larger tumor size, older age, male sex, extrathyroidal extension, and higher serum calcitonin and CEA levels also affect survival in univariate analyses. In line with previous studies, we found that independent prognostic factors other than grade identified on multivariate survival analysis included age, AJCC eighth edition prognostic stage group, margin status, postoperative serum CEA, postoperative serum calcitonin, and vascular space invasion.^3,11,18,19^

Given the robust independent prognostic value of the IMTCGS grade, we advocate including this histologic grade in risk stratification and clinical decisions for MTC. Patients with high-grade MTC have an increased risk of locoregional and distant metastasis, and therefore may benefit from early lateral neck lymph node resection, close follow-up, low thresholds for cross-sectional imaging, and careful work-up for distant metastasis. Furthermore, we recommend including histologic grade as a datapoint in the assessment of any future clinical trials of adjuvant therapy (eg, those for RET inhibitor therapies) as it is likely that adjuvant therapy will have greatest benefit in high-grade tumors.

Somatic mutation testing is increasingly being performed to assess the potential benefit of RET inhibitor therapy in advanced MTC, and one limitation of this study was that we did not have data to correlate the grade with the type of somatic mutation. It is noteworthy that in a previous investigation of grading on the basis of MI, Ki67, and necrosis in a small series of 44 sporadic MTCs, there was no correlation between these factors and *RET* or *RAS* somatic mutation status.^12^ Although assessment of mutation status may further refine risk stratification in MTC, additional multicenter studies will be required to further evaluate the relationship between mutation status, grade, and outcome.

Given that tumor necrosis may be present only focally in MTC, we advocate for generous sampling of tumors in resection specimens to accurately assign histologic grade. Additionally, we acknowledge that grading may not be as accurate in smaller specimens such as biopsies that may not capture focal tumor necrosis, although this should be clarified in future studies.

In conclusion, in this multicenter study, we propose and validate the IMTCGS based solely on MI, Ki67 proliferative index, and tumor necrosis, which is analogous to the grading of NETs of other organs. To subdivide large cohorts into meaningful risk groups, we demonstrate that a two-tiered system that defines high-grade MTCs as tumors with at least one of the following three features: MI ≥ 5 per 2 mm^2^, Ki67 proliferative index ≥ 5%, and/or tumor necrosis, identifies MTCs with a profoundly greater risk of progressive and fatal disease. However, we emphasize that mitotic rate and Ki67 proliferative index are continuous variables, and prognosis worsens as the proliferative activity of a tumor increases. Therefore, the precise mitotic rate and Ki67 proliferative index should be recorded in the pathology report for all MTCs to help further refine risk stratification.
